# Navigating strategies used to manage fatigue by patients undergoing hemodialysis: a qualitative exploratory design

**DOI:** 10.1186/s12882-025-04140-0

**Published:** 2025-05-05

**Authors:** Zakariya Al-Naamani, Kevin Gormley, Helen Noble, Olinda Santin, Omar Al Omari, Huda Al-Noumani, Norah Madkhali

**Affiliations:** 1 Nephrology and Palliative Support, Medical City for Military and Security Services School, Ministry of Defense, Al-Khoudh, P.O. Box 721, Muscat, 111 Oman; 2 Nephrology and Palliative Support, College of Nursing, Sultan Qaboos University, Al-Khoudh, P.O. Box 66, Muscat, 123 Oman; 3 Nursing and Health Sciences, College of Nursing and Midwifery, Mohammed Bin Rashid University of Medicine and Health Sciences, Dubai, United Arab Emirates; 4 School of Nursing and Midwifery, Queen’s University Medical Biology Centre, Belfast, BT9 7BL UK; 5 Mental Health, College of Nursing, Sultan Qaboos University, Al-Khoudh, P.O. Box 66, Muscat, 123 Oman; 6 Adult Health and Critical Care, College of Nursing, Sultan Qaboos University, Al-Khoudh, P.O. Box 66, Muscat, 123 Oman; 7 Cancer Nursing and Palliative Care, College of Nursing, Jazan University, Jazan, Saudi Arabia

**Keywords:** Fatigue, Self-management, Experience, Oman, End-Stage kidney disease, Hemodialysis

## Abstract

**Background:**

Fatigue among patients with end-stage kidney disease (ESKD) undergoing hemodialysis significantly impacts quality of life and anticipated treatment results. This study explores how Omani patients undergoing hemodialysis manage their fatigue.

**Methods:**

A qualitative design was employed. Data were collected from 25 participants through semi-structured interviews, which were transcribed verbatim and analyzed using thematic analysis. Data management, memo creation, and annotation were completed using NVivo 11.

**Results:**

Findings highlighted one main overarching theme, Self-initiated management strategies, outlining the coping mechanisms patients used to reduce fatigue and adapt their daily activities to its ongoing presence. This overarching theme encapsulates six subthemes that describe patients’ self-initiated strategies to manage fatigue, which include (1) self-regulating weight gain and negotiating accumulated fluid removal with healthcare providers (HCPs); (2) Increased appetite and desire for rest; (3) expanding self-awareness for change; (4) engaging in regular physical activity; (5) seeking deeper understanding and support from others and (6) immersing in faith and religious practices.

**Conclusions:**

The study findings emphasize that patients used several management strategies to manage both physical and mental fatigue and improve their quality of daily living. Although these techniques used to manage fatigue by patients were helpful, healthcare professionals must provide a holistic approach to support the patient’s self-initiated fatigue management strategies. Therefore, further studies would be required both within nationally and internationally to validate study findings, and find methods to promote the positive coping techniques used.

**Clinical trial number:**

Not applicable.

**Supplementary Information:**

The online version contains supplementary material available at 10.1186/s12882-025-04140-0.

## Background

Chronic kidney disease is a global health concern as the prevalence of CKD increased by almost 90% from 1990 to 2016. Incidence of end-stage kidney failure (ESKD) cases increased by 118.7% between 2000 and 2019, leading to a rapid rise in demand for renal replacement therapy, including hemodialysis [1, 2]. This significant increase is attributed to rises in the prevalence of diabetes and hypertension, particularly among the Gulf Cooperation Council (GCC) states [1]. In Oman, statistics show an overall increase in the prevalence of patients with ESKD undergoing hemodialysis from 35 to 2023 patients between 1983 and 2018, respectively [3].

Hemodialysis plays an important role in managing ESKD and sustaining the lives of patients. Despite the therapeutic value this mode of treatment does frequently result in negative effects on patients physical, psychological, and social well-being [4, 5]. Patients undergoing hemodialysis often experience debilitating symptoms that impair their activities of daily living, causing physical and mental fatigue, and a reduced quality of life [4, 6]. Fatigue is one of the most frequently reported side effects of hemodialysis, with an estimated prevalence ranging from between 49 and 92% [7–9]. A recent study in Oman reported that patients undergoing hemodialysis perceive fatigue as an inevitable symptom and cause physical and mental exhaustion in their daily lives [6]. The definite causes of fatigue among patients receiving haemodialysis are not well understood [7]; however, several contributors that influence the severity of fatigue have been identified, for example restless legs syndrome, poor sleep quality [10, 11], itching, chronic anemia, depression, bone and joint pain [12, 13]. Additionally, comorbidities and dialysis inadequacy are also associated with fatigue severity [14].

Despite the advancement in hemodialysis technology and care delivery, patients undergoing hemodialysis have limited fatigue management strategies or options to manage their hemodialysis-related symptoms effectively, which negatively impacts their quality of life and increases mortality risks [6, 15–18].

Treatment of fatigue among patients undergoing hemodialysis is complex, as the causes are multidimensional [4, 19]. Fatigue remains frequently under-recognized, poorly managed and perceived as a normal and expected consequence of ESKD and hemodialysis [6, 9, 20]. Thus, treatment for fatigue is limited and care focuses mainly on correcting patients’ physiological variables such as anaemia and fluid volume levels through hemodialysis [4, 21].

Common pharmacological interventions used to help patients feel less fatigued include erythropoietin (EPO), L-carnitine and vitamin C supplementation [22–24]. Artom et al. (2014) argue that pharmacological interventions are not significantly effective in preventing and treating fatigue [23]. Non-pharmacological interventions such as increased exercise (physical and relaxation), proper nutrition, and aromatherapy may also contribute towards reducing fatigue severity and improving patients’ psychological wellbeing [25, 26]. The effectiveness and generalisability of these interventions and their long-term impact on fatigue reduction needs further investigation [23, 25, 26]. Recent evidence suggests that a better understanding of fatigue-related management and its effectiveness to improve fatigue management and quality of life for patients undergoing hemodialysis is warranted [6, 19, 21]. Therefore, the aim of this study is to explore how patients undergoing hemodialysis manage their fatigue to improve physical and psychosocial well-being. Understanding patient self-initiated fatigue management strategies helps provide better care and holistic approach based on patient preferences.

## Methods

### Study design

An exploratory qualitative research design was used to gain understanding as to how patients undergoing hemodialysis manage their fatigue The exploratory qualitative approach is employed to acquire new, comprehensive insights regarding the diverse methods by which a phenomenon is demonstrated and the fundamental processes that underlie it, as described by participants [27]. This is particularly necessary in cases where the topic of investigation or phenomenon has limited coverage and is not fully understood in the literature.

### Sample and setting

Study participants were recruited from two hemodialysis centers in Oman. The centers offer dialysis four times a day: morning, early afternoon, late afternoon, and evening. Participants were required to attend three sessions per week, each lasting four hours.

Eligibility criteria included Omani adult patients aged 18 years or older; diagnosed with ESKD; and receiving hemodialysis for at least six months with a Likert scale fatigue score ≥ 1 (0 (no fatigue) to 10 (severe fatigue)). Six months of hemodialysis was considered sufficient for patients to adapt to the hemodialysis treatment process. This time window is important because during the first phase of hemodialysis, patients undergo a critical transition and experience significant physiological and functional changes that may affect their perception of fatigue [28]. Participants were excluded if they had been diagnosed in their medical records with cancer, HIV, severe heart failure, anxiety or depression because these patients may experience fatigue unrelated to hemodialysis.

### Ethical approval

The Ministry of Health’s Ethics Committee in Oman approved this study (MOH/CSR/18/9002). The research followed the principles outlined in the Helsinki Declaration (version 2013), and all analyses were conducted anonymously using coded numbers, which are not linked to participants’ real details.

### Data collection

An interview guide based on Price’s (2002) framework was developed through a literature review, expert consultation, and was tested with three patients undergoing in-center hemodialysis to ensure that the questions were clear and relevant to exploring self-management strategies for hemodialysis-related fatigue (Table 1). Minor changes were made related to cultural aspects. These interviews were excluded from the final analysis [29].

After ethical approval was received, a senior nurse who had direct contact with patients approached potential participants during treatment to inquire about their willingness to participate in the study and provided them with a patient information sheet containing detailed information about the study.

Of the 76 potential participants who met inclusion criteria, 25 agreed to participate in the semi-structured interviews. The principal investigator (ZA) contacted participants and met with them at a mutually agreed location and time and obtained consent. The interview process began with informal conversation to build rapport, establish trust, and allow for flexibility in the interview [30]. After breaking the ice and building rapport, the researcher started the recording session, and began with general and minimally invasive questions, ensuring that no identifying information was captured [31], followed by more probing questions to obtain detailed information that would ultimately advance the research objectives.

All interviews were conducted on a one-by-one basis, lasted between 30 and 80 min. Most took place in a convenient, quiet and private room within the hemodialysis center before their session, except for 5 interviews, which were conducted during the hemodialysis session. They were recorded and transcribed verbatim, and field notes were taken during the process [32, 33]. Data was stored using a secure and encrypted computer to ensure data security and participant privacy.

**Table 1:** Framework for semi-structured interview questions

Type of question		Questions	Probing questions
Warm up		Could you introduce yourself? Could you tell me about the symptoms that you have experienced since you started hemodialysis?	What is your name, marital status, age, number of children, area you work in, education, how long you have been receiving hemodialysis?
Experience	Knowledge/beliefs	Could you tell me how you manage your fatigue and what approaches you take?	What do you know about self-management approaches? What are the benefits of self-management approaches in terms of your health and reducing or managing your fatigue from your point of view?
	Actions/feelings	From your experience, can you tell me what you usually do when you experience fatigue?	How do you manage your own fatigue? Can you provide examples of how you manage your own fatigue? What is your role in reducing your own fatigue?
	Feelings/ attitudes/beliefs	What do you think motivates you to cope with fatigue?	Do you think your family and friends understand your fatigue? How? Why? Do you think they have enough information to help you manage or reduce your own fatigue?

### Data analysis

The semi-structured transcripts were systematically examined using the six-step approach to thematic analysis, which consists of (1) familiarization with the data (reading and re-reading), (2) generating initial codes, (3) searching for themes, (4) reviewing themes, (5) defining and naming themes, (6) producing the results [34].

During the first phase of data management, all interviews were transcribed verbatim. The researcher (ZA) translated interviews conducted in Arabic to English. To ensure translation accuracy, a quarter of the translated interviews were randomly chosen for thorough review by other researchers fluent in both Arabic and English (HS & NM). The raw data was managed with NVivo 11, which made it easier to organize the dataset and create memos and annotations to show the relationships between codes and themes.

Descriptive coding entailed reading the transcripts multiple times to capture a precise representation of the raw data and develop an overall understanding of emerging concepts within the descriptions [33]. The text was then divided into manageable meaning units while keeping the original content intact. These condensed meaning units were then assigned codes and organized into categories based on similarities and possible relationships. The researcher conducted additional analysis within each category to identify overarching themes, interpretive themes, and subthemes.

**Fig. 1:**
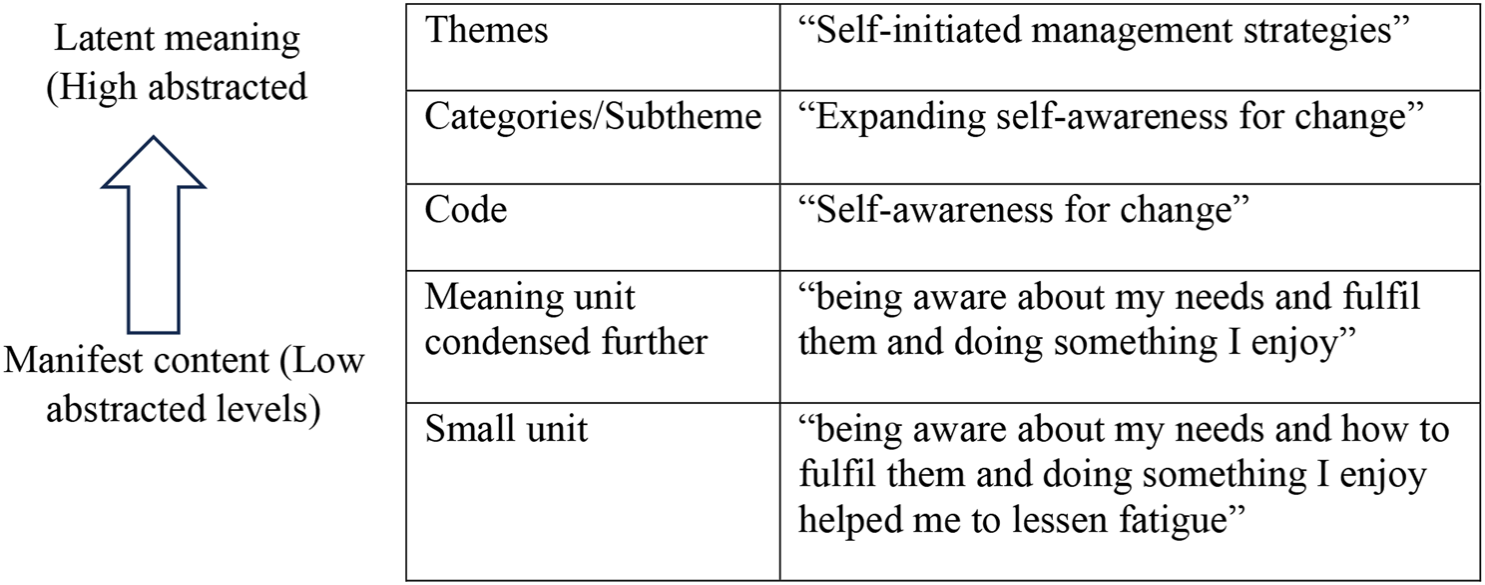
Example of the data reduction process

To visualize and interpret the latent content, the transcripts were read several times to accurately represent the meaning of the raw data. The text was then divided into small meaning units, which were condensed while retaining the original meaning and labeled as codes. Finally, similar and related codes were categorized, resulting in subthemes and overarching themes (Fig. 1). To ensure the analysis’s quality and rigor, an expert panel (KG, HN, and OS) reviewed the findings in detail to validate the accuracy and authenticity of the interpretations [35].

### Trustworthiness

Throughout the analysis process, the researchers followed the four criteria proposed by Lincoln and Guba: credibility, transferability, confirmability, and dependability to ensure the quality of the data and preserve its credibility [25]. In qualitative research, these criteria are thought to be necessary to ensure the validity of the data interpretation and the accuracy of the drawn conclusions.

## Results

The patients’ sociodemographic details are shown in Table 2. The duration of patients’ hemodialysis treatment ranged from 1 to 17 years, with an average age of 41.32 years. Of the patients, 44% were a full-time workers, and 68% were married.

**Table 2:** Patients’ sociodemographic data

**Ethnicity**		***N***
Omani		25 (100%)
**Age**		**Mean (range)**
Total sample (*n* = 25)		41.32 (23–62)
Female (*n* = 9)		41.37 (23–49)
Male (*n* = 16)		41.30 (27–62)
**Education**		***N***
Bachelor degree		7 (28%)
Diploma degree *		6 (24%)
School degree **		12 (48%)
**Marital status**		***N***
Single		7 (28%)
Married	Average No. of children = 3.70 (0–9)	17 (68%)
Divorced		1 (4%)
**Employment status**		***N***
Full time		11 (44%)
Retired		7 (28%)
Unemployed		7 (28%)
**Living arrangement**		***N***
With own family		16 (64%)
With Parents		6 (24%)
With Husband’s family		2 (8%)
With son***		1 (4%)
**Dialysis duration/yrs**		**Mean (range)**
Total sample (*n* = 25)		5.52 (1–17)
**Timing of dialysis sessions**		***N***
Morning		6 (24%)
Early afternoon		8 (32%)
Late afternoon		5 (20%)
Night		6 (24%)

*Diploma degree refers to higher education (post-secondary school)

**School degree refer general education (Grade 1 to 12)

*** With son indicates that the participant resides in their son’s household along with his family

The thematic analysis of the study data revealed an overarching theme, **Self-initiated management strategies**, which outlined the coping mechanisms patients used to reduce fatigue and adapt their daily life activities to its ongoing presence. This theme captured six subthemes (Table 3), which illuminate these strategies as follows: (1) self-regulating weight gain and negotiating accumulated fluid removal with HCPs; (2) Increased appetite and desire for rest; (3) expanding self-awareness for change; (4) engaging in regular physical activity; (5) seeking deeper understanding and support from others and (6) immersing in faith and religious practices.

**Table 3:** List of emergent theme and subthemes

Overarching theme	Subthemes
Self-initiated fatigue management strategies	1. Self-regulating weight gain, and negotiating fluid removal 2. Increased appetite and desire for rest 3. Expanding self-awareness for change 4. Engaging in regular physical activity 5. Seeking deeper understanding and support from others 6. Immersing in faith and religious practices

### Self-regulating weight gain, and negotiating fluid removal

A common strategy used by all patients to reduce fatigue was to manage weight gain between hemodialysis sessions by minimizing their fluid intake. Most patients sought to moderate their fluid intake to make the hemodialysis sessions more tolerable. Although weight gain was expected due to their inability to excrete fluid, patients reported that it made them feel unwell and many resorted to strategies to reduce its impact.

*… I feel fine as long as I maintain my extra weight at no more than 3.5 kg above [normal].* (Phase 1: NHC: P02)

The negative feelings associations with extra weight were most prevalent during non-hemodialysis days, as extra weight affected patients’ physical and psychological wellbeing and their quality of life.

*If I don’t do dialysis for two days*,* the amount of fluid in my body will be greater [than usual] and I feel very exhausted…* (Phase 1: BHC: P22).

In addition to self-initiated weight management strategies such as limiting oral fluid intake, patients who felt they had accumulated extra weight often negotiated with hemodialysis staff over the amount of fluid that could be safely removed on a particular day depending on their perceived level of tolerance. In such cases, the patients negotiated the remaining fluids to be removed during the hemodialysis session. This negotiated fluid removal strategy led to less severe Post Dialysis Fatigue (PDF), which in turn helped patients regulate their weight before the next session, as this participant explained:

*[I]f I drain more than 4 kg during my dialysis session*,* I feel very exhausted… For example*,* if I’ve gained 5–6 kg extra weight*,* I ask the staff to remove only 4 kg*,* and the remaining 1–2 kg will be removed next session. At home and before next session*,* I make sure to drink and eat less*,* and sometimes I fast for one day to make sure I don’t gain much weight.* (Phase 1: NHC: P05)

### Increased appetite and desire for rest

Most participants sought to eat, rest and sleep immediately after hemodialysis to recover from the fatigue. Most reported feeling ‘an unusual hunger’, which further aggravated the feeling of PDF. When asked about this post hemodialysis, most patients reported that they usually had a good appetite and felt the need to eat post hemodialysis and frequently ate a large meal to boost their energy levels before going to sleep.

*I arrive home at 1 p.m. I feel like I am starving and have no energy to do anything. That is why I take a heavy lunch before I sleep… (Phase 1: BHC: P15)*.

All participants emphasized the importance of resting and sleeping directly after hemodialysis to manage PDF and to be able to resume daily activities. While the need for sleep appeared to be universal, the recovery time they required varied from patient to patient. Because the recovery time from PDF was unpredictable, the sleep they required also varied. Some participants needed only a less hours of sleep while others required several hours, sometimes extending to the next day before they felt fully recovered.

*I can’t say precisely because it depends on fatigue. I feel mostly fine when I sleep for two to four hours after the sessions. Sometimes, I need to sleep the whole day to feel fine… (Phase 1: BHC: P23)*.

The amount of time patients needed to rest after hemodialysis seems to depend on the severity of their PDF. The findings from this study clearly indicate that those with less severe PDF symptoms needed shorter periods for rest compared to those who experienced more severe symptoms following hemodialysis. The findings revealed that patients with less severe symptoms recovered more quickly and were able to involve themselves in and effectively manage their daily obligations and responsibilities. Participants in this category described PDF as a ‘slight ordinary tiredness’ to which they had become accustomed and which they had integrated into their daily life routine. Some patients suggested that the reason they experienced less severe PDF was because they were more actively engaged in daily life activities and socializing with families.

*… after dialysis sessions. I go directly to visit my mum and have food with her because I feel hungry. After that, I go to the farm and do some work… (Phase 1: NHC: P04)*.

### Expanding self-awareness for change

Most participants acknowledged that understanding their current strengths, weaknesses and emotions related to the illness would help them overcome the challenges associated with hemodialysis. As a result of this increased self-awareness and proactivity, some patients were able to continue to work full-time while receiving hemodialysis three times per week.

*being aware about my needs and how to fulfil them and doing something I enjoy helps me to lessen fatigue*,* feel psychologically comfortable*,* and enjoy my day. (Phase 1: BHC: P11)*

Being self-aware helped some to identify life stressors which influenced fatigue severity. As they became more aware of their mental and physical wellbeing through introspection and reflection, such patients become more able to take active steps to restore their real self-identity, which the illness had somewhat eroded. One aspect of this introspection was learning more about their emotions and how to deal with them. This ability to examine their thoughts and feelings and rebuild their self-identity and modify their behaviour had a positive effect on their physical and mental health.

*I was having negative thoughts, felt angry easily, and my mood was unpredictable. I think, if I hadn’t begun to notice myself and learn to change these feelings, I would be a negative person by now.… Now, I don’t feel angry, and being calm saved my energy level and I feel relaxed and comfortable (Patient smiles). (Phase 1: NHC: P01)*

The study findings revealed that emotional intelligence was related to social integration with others. Those who were more emotionally intelligent (i.e. more aware of their thoughts, the responses of others and how they could be positively managed) were more likely to try to adopt a positive attitude about their condition despite their fatigue, and therefore more likely to report a level of social integration similar to that which they enjoyed pre-hemodialysis. Such participants reported feeling self-motivated, sociable with others, optimistic about their lives, and intentionally sought to make behavioural changes, especially those they believed could improve their physical and psychological wellbeing.

*I realize that if I don’t motivate myself and make myself optimistic, I feel emotionally tired and more fatigued. (Phase 1: BHC: P13)*

Conversely, patients who tended to have negative thoughts appeared to gravitate towards negative attitudes, which appeared to increase the severity of their fatigue. These patients had poorer self-management and behavioural skills, and reported reduced motivation to address their negative emotions and behaviours. The researcher also noted that most of these patients gave up easily and this negativity translated to them presenting as more passive about seeking change to their lives and how they handled events such as emotional meltdowns (things become unbearable) and aggression towards others,

*Everything changed since I started hemodialysis. Even my husband told me once, ‘You become aggressive and irritated easily for no reason sometimes.’ I don’t know what to do to change such bad behaviours. (Phase 1: BHC: P17)*

### Engaging in regular physical activity

Participants reported that physical exercise helped them to reduce the severity of fatigue and increase their muscle endurance, thereby increasing their ability to manage daily life activities.

*If I stop doing exercises for a while, I feel fatigue, pain in my legs and psychologically overwhelmed. (Phase 1: NHC: P04)*

Regular physical activity helped patients improve their physical fitness and maintain optimal health. It also prevented them from developing complications that could contribute to their fatigue, such as muscle weakness and eventual atrophy. Those patients who realized the benefits of exercise continued to be motivated and maintained regular physical activity despite the experience of fatigue. In fact, those who were physically active reported that they felt energetic and suffered less pain.

A major motivation for patients, apart from countering fatigue, was their belief that regular exercise would reduce their risk of cardiovascular disease and diabetes that would further aggravate negative consequence of kidney disease and fatigue. In addition to the physiological benefits, participants acknowledged that regular physical activity helped reduce stress and improve their mood.

*I must do some activities to maintain fitness*,* muscle strength*,* and if I don’t go for a walk*,* I feel sluggish*,* uncomfortable and more fatigue… I do exercise also because I don’t like to be at risk for diabetes or heart problems*,* and you know we’re vulnerable for such things. (Phase 1: BHC: P22)*

Most participants expressed that less strenuous forms of exercise were more suitable. While walking was the form of exercise favoured by most hemodialysis patients, some participants preferred even less strenuous group exercises such as Zumba and yoga, both of which are based upon a program of choreographed movements designed to promote fitness and wellbeing. It was interesting to observe that most participants felt motivated and encouraged when engaging in group exercises, or when a relative or friend accompanied them to an exercise session.

*I realized doing Zumba with the group motivated me and I was socially engaged as compared with being alone at home… (Phase 1: BHC: P17)*.

Most participants reported that they performed their exercises on non-dialysis days as this helped to improve their energy levels and psychological wellbeing.

*I go for a walk on the beach for at least 30 min on off-dialysis days, and sometimes I swim in the sea. (Phase 1: BHC: P22)*

*Almost four times per week I go for a walk for at least half an hour, and sometimes I go cycling, which helps me to be energetic and feel less stressed. (Phase 1: NHC: P04)*

While most patients advocated regular exercise, they were aware of the limitations they had come to accept as a consequence of their disease, condition and treatment. Participants said that they were incapable of involving themselves in strenuous exercise that required physical and mental exertion, such as football or ping-pong and athletics. Many participants reported that they had stopped playing team sports because they exhausted easily and were concerned about the potential risk of injury, especially to the fistula site. Others expressed concern that strenuous exercise could increase the risk of unpleasant symptoms such as pain, dyspnoea and heart-related problems.

*[M]ost exercises are not appropriate for us [patients on hemodialysis] and I think patients on dialysis should avoid heavy exercises, because they might deteriorate, and have a lot of problems or even heart complications. (Phase 1: BHC: P19).*

Findings revealed that participants who engaged in minimal physical activities, or those who had a sedentary lifestyle, appeared to suffer from more fatigue that those who, despite the difficulties, endeavoured to keep active. Indeed, the findings indicate that the less active patients tended to be less motivated and less socially engaged compared to their more active peers.

The less active participants assumed that engaging in exercise would increase their feelings of fatigue, hence they preferred to use their energy to complete house chores, rather than ‘wasting time’ on physical exercise.

*[I]t’s difficult to do exercise because I’ve no energy and spend most of my time at home.… and I think doing household chores is enough for me*,* so no need to do exercise.* (Phase 1: BHC: P07)

### Seeking deeper Understanding and support from others

The study participants echoed a view that being understood and supported by others was essential to managing fatigue and maintaining a sense of normality or balance in life. To this end, hemodialysis patients sought support from their families, siblings, hemodialysis centre staff and, for those still employed, from their colleagues in the workplace. Participants reported that having support networks helped them to effectively manage fatigue in their daily lives.

*Honestly*,* with this treatment I wouldn’t have been able to move on in my life without my family’s support. (Phase 1: BHC: P07)*

The family emerged as the primary and greatest source of support for patients receiving hemodialysis, who viewed it as essential for maintaining family unity and relationships. The findings indicate that such support was most appreciated when it involved help with tasks that patients had previously failed to accomplish due to severe fatigue and other challenges.

*My family’s support and understanding helps me a lot to cope with my treatment and fatigue*,* especially tasks which I can’t do. (Phase 1: BHC: P14)*

The study revealed that many spouses cared for the needs of their sick wives or husbands. Participants reported that spouses often go to great lengths to create a comfortable and relaxing atmosphere to help the patient enjoy a good rest and sleep. This extra care was pronounced when patients arrived home from hemodialysis sessions. Over time, family members, especially spouses, became familiar with the patient’s fatigue and associated emotional problems. This knowledge makes families much more able to meet the patient’s needs, which helped them manage their physical and mental fatigue.

*… When I [patient] feel burnout when I can’t fulfil my responsibility and am in a bad mood*,* my husband will usually take me out for a walk on the beach. We talk and share stories*,* which really help me to feel better and divert the negative thoughts in my mind that I can’t get rid of it without his [her husband’s] support.* (Phase 1: BHC: P20)

Most participants felt that being with friends helped to create an enjoyable and relaxing atmosphere. The findings indicate that friends, especially close ones, motivated hemodialysis patients to exercise and engage in social activities that enhanced their psychological wellbeing and thus levels of fatigue.

*I feel motivated to do exercises with them [friends] and we have fun while we are doing exercises. Sometimes*,* I travel with them on short trips…* (Phase 1: BHC: P08).

Participants in full-time employment spoke about the support and the understanding they received from their managers, which helped them to manage fatigue and their work responsibilities. The support patients received in the workplace took various forms. In some cases, the patient’s manager allowed them to rest when they were tired. Others reported that their managers gave them enough time to accomplish their assigned tasks, while in other cases managers helped patients to adjust and coordinate between their work and hemodialysis sessions. Having an understanding manager who accepted and made accommodations for their medical needs affected employed patients positively by putting their minds at rest.

*At work, they [the manager and co-workers] are very supportive and helpful. They help me to adjust between my treatment and work…* (Phase 1: BHC: P10).

*… This supportive working environment decreased my stress. Because their support calmed me psychologically because I was worried about losing my job… (Phase 1: BHC: P24)*.

The most significant support HCPs offered patients was medical management of their condition. Specifically, HCPs worked to rectify abnormal blood investigation values and prescribed medications such as iron supplements or epoetin injections to improve haemoglobin levels and other medications to eliminate or reduce unpleasant symptoms such as itching, which contributed to fatigue.

*[I]f the haemoglobin falls below 9, the doctor prescribes medications during my session such as iron and epoetin injections.* (Phase 1: NHC: P01)

This participative form of care and support led most participants to feel that there was a shared responsibility between medical staff and patients in maintaining normal blood values to avoid unpleasant symptoms that contributed to fatigue. They were eager to receive the results of their blood work from clinical staff, such as blood phosphorus and haemoglobin levels, because these were deemed essential to allow them to modify their diet and fluid intake after each session. In fact, patients took deliberate steps to avoid abnormal results that might cause low energy levels or contribute to the feeling of fatigue.

*One of the things I do to get energy and feel less fatigue is eating what I like*,* but it is very important to know my blood results on a regular basis because if the result is abnormal*,* I need to avoid certain foods…(Phase 1: BHC: P11)*.

### Immersing in faith and religious practices

All the patients who took part in this study were self-reported as being Muslim, and all considered a faith in Allah to be an essential coping and motivational strategy in the management of fatigue. Islamic religious practices such as prayer were adopted by patients as complementary and alternative treatment modalities during their ill health. Indeed, patients’ absolute faith in Allah and the Islamic commandments empowered them to accept their illness and the treatment process, which in turn helped to reduce mental fatigue.

*As a Muslim*,* I believe that this is a matter of judgment and fate… When I feel fatigued*,* I pray to Allah to give me strength and my faith in Him keeps me motivated during my day to do my tasks. (Phase 1: BHC: P19)*

Participants reported feelings of gratefulness to Allah because they were still alive, and blessed because they had access to hemodialysis, knowing that others were not so fortunate. A sense of spirituality and religious belief seemed to motivate participant to commit to their hemodialysis regimen and also empowered them to cope with fatigue. Other participants reported an optimism about life, even though they clearly had poor health.

*… Yeah, fatigue is present but with the hemodialysis treatment and my faith in Allah, I feel fine as long there are no other complications. (Phase 1: BHC: P07)*

*… I [patient] thank Allah for his blessing because having kidney failure isn’t the end of life and we’ve a treatment while other patients don’t. (Phase 1: NHC: P01)*

Most participants felt that praying in the mosque provided spiritual support, calmness and peace of mind. Visiting and praying in the mosque also offered a community context, providing a means of maintaining social contact with families and neighbours. For some, walking to the mosque was a form of exercise and helped to improve energy levels. In summary it seems there were benefits to be had from having spiritual belief and the process of demonstrating that belief through attending the mosque that helped them to enhance physical and psychosocial wellbeing.

*I pray most of the time in the mosque*,* where the spiritual atmosphere helps me to relax and feel an inner peace… Sometimes*,* we [the people in the mosque] gather and share stories and news… Also*,* one of the things that helps me a lot with my fatigue is when I walk to the Mosque. The Mosque is about 500 m from my house… This is really exercise for me!* (Phase 1: BHC: P14)

Some patients opted to read or listen to the Quran during hemodialysis sessions in order to relax, ‘calm their soul’ and avoid boredom. Reading the Quran helped patients to cope with difficulties resulting from the treatment process and the associated fatigue.

*… I read the Quran to relax and sleep*,* especially when I’m tired*,* or I can’t sleep due to disturbing thoughts that keep me awake. (Phase 1: NHC: P04)*

## Discussion

The study findings reveal that patients undergoing hemodialysis mostly manage their daily fatigue experience independent from healthcare staff or family and friends or both because they received little professional support from healthcare providers to help manage their fatigue. In the absence of such help, many patients reported utilizing various self-initiated techniques, to identify their own workable strategies to deal with fatigue. Similar findings were highlighted in several studies that indicate HCPs provide limited support for patients undergoing hemodialysis to manage fatigue, and mainly focus on providing medical support [4, 36–38].

It is worth noting that although the study’s focused to explore chronic fatigue among the hemodialysis population, post-dialysis fatigue (PDF) was often highlighted by participants as a predominant experience. This can be attributed to the fact that the severity and sudden onset of PDF occur immediately after each dialysis session, making it a more noticeable and challenging aspect of fatigue to manage. Therefore, understanding how patients undergoing hemodialysis manage their fatigue, including PDF, is crucial for developing effective self-management strategies to enhance patients’ well-being [4, 17].

The study found that patients undergoing hemodialysis depend on several self-initiated strategies to manage fatigue including self-regulating weight gain, desire for increased appetite and rest, expanding their self-awareness for change, engaging in regular exercise, seeking deeper understanding and support from others, immersing in faith and religious practices. It is worthily to mention that 44% of the study participants were employed on a full-time basis, which may suggest that those who implement fatigue management strategies exhibit a higher level of functionality at baseline relative to the broader population of patients undergoing hemodialysis.

One of the strategies patients used to self-regulate their weight (i.e. patients’ weight with extra fluid in their body) and manage fatigue was to minimize their fluid intake. Managing weight and fatigue is a constant struggle for hemodialysis patients as they get more fatigued if too much weight is taken off, which can affect their quality of life [39]. Patients therefore negotiate with HCPs, based on their perceived levels of tolerance, the amount of fluid to be removed (from actual weight) during some hemodialysis sessions; the remaining fluid is removed during the subsequent session. This, they believe, helps them manage their post-dialysis fatigue (PDF). However, the constant struggle to manage weight and fatigue can be harmful as the remaining fluid can overload the patient’s heart and predispose them to cardiovascular problems [40]. This suggests a lack of awareness amongst patients about the long-term impact of the remaining fluid, non-adherence to fluid intake restrictions and dietary negotiation. Glyde et al. (2019) found that hemodialysis patients had a good understanding of the short-term effects of adjusting fluid removal during dialysis sessions but had a poor understanding of the long-term consequences of regularly doing so [41]. Therefore, patients need to be provided with adequate and regular information on the importance of adequate fluid intake in weight management and the detrimental effects of fluid removal during dialysis.

Patients undergoing hemodialysis also manage their fatigue by eating more after hemodialysis sessions, rest and sleep. However, the prevalence of non-adherence to the recommended dietary regimen presented as a global concern observed in 47.3–72.5% of hemodialysis patients [42]. In an integrative review, associated factors for diet adherence among hemodialysis patients was investigated, they found that the failure of hemodialysis patients to adhere to their recommended diet worsened their fatigue and caused cardiovascular problems [43]. Furthermore, a systematic review conducted among ESKD patients showed that poor diet and fluid intake compliance were significantly associated with depressive symptoms and insufficient dietary support [44]. Factors that contributed to this outcome included lack of knowledge among patients and their families, presence of chronic illnesses, lack of family support, depressive symptoms, and sociocultural challenges, such as eating habits at social gatherings. As a solution to non-adherence, Stevenson et al. (2018) recommended multidisciplinary collaboration to deliver meaningful nutritional advice [45]. Multidisciplinary teams can help sustain patients’ motivation and enable them to change their dietary behaviours.

Another strategy adopted by some patients to manage their fatigue was becoming aware of the emotions and behaviours which contributed to their physical and mental fatigue. These findings concur with several studies, that found patients were able to control their fatigue by understanding their body’s capabilities and developing strategies to save or produce energy accordingly [21, 46]. This mindful approach, and acknowledging their abilities and limitations, helped patients to avoid overdoing things, enabled them to become resilient and compassionate toward themselves and improved their ability to handle their daily activities [9, 21].

Seeking deeper understanding and support from others is another strategy patients used to manage fatigue. The assurance of support from significant others (parents, siblings, spouses and children) helps them cope with their physical and mental fatigue. This finding aligns closely with that of other studies [9, 38, 47]. When Alshammari et al. (2023) evaluated the relationship between fatigue and family support among hemodialysis patients, they found that a higher level of family support helped reduce fatigue [48]. This is fully endorsed by the findings of the present study, which demonstrated that married patients, particularly those with older children, displayed less fatigue than patients with young children. Because older children are more independent, they are able to provide support and help their sick parent in managing their daily life activities. In contrast, young children, who are dependent, require intensive care and support from their parent. It is important, therefore, to provide adequate information and professional input to the family so as to help patients manage fatigue in their home environment and subsequently improve their ability to carry out their daily activities based on their cultural context [49].

Many patients reported that they immersed themselves in faith and religious practices as a strategy to deal with fatigue. The importance of religion to individuals coping with psychological stress during hemodialysis has been highlighted in several international studies [50–53]. This seems to be especially the case for Islamic beliefs and practices, which have been known to serve multiple functions for patients dealing with fatigue and its psychosocial impact. A study conducted in Jordan showed that Muslim patients receiving hemodialysis used spiritual and religious practices as a coping mechanism to resolve personal and psychological challenges [52]. Therefore, positive and healing beliefs have been linked to improved physical and psychosocial wellbeing [54, 55].

### Strength and limitation

The study’s strengths include a well-defined population and a relatively large sample size, which provided valuable insights into fatigue from the participants’ perspectives. Additionally, there are certain limitations, such as the timing of data collection. The depth of the data acquired and the freedom of patients to express themselves may have been impacted by the fact that some patients were interviewed during dialysis sessions. Translation of some information from Arabic to English may have hindered data interpretation. Generalization of the findings may be limited due to differences in the cultural background and hemodialysis services across countries. The exclusion of patients with depression may also decrease generalizability of the findings, as depression is common in this population. The interpretation of data also may have been impeded by the translation of certain information from Arabic to English. The findings’ generalizability may be restricted by variations in cultural backgrounds and hemodialysis services between countries.

### Implication for nursing education, practice, and research

Fatigue in patients undergoing hemodialysis is a multifaceted phenomenon influenced by both physical and mental causes. Consequently, healthcare personnel must evaluate and manage patients beyond conventional hemodialysis treatment and do regular holistic assessments to identify potential patient resources based on their cultural backgrounds to assist them in coping with the inevitability of hemodialysis-related fatigue. Additionally, HCPs should also have access to specialized educational and training programs for identifying and managing the complex symptoms of ESKD, including fatigue. More research into patient-initiated fatigue management strategies is needed, as this could provide insights into prospective fatigue-specific contextual interventions.

## Conclusion

Fatigue among Omani patients undergoing hemodialysis receives less attention from healthcare providers than, and hence, patients depend on self-initiated management-based strategies. Understanding how patients manage their fatigue could result in more effective management strategies and earlier assessments, as it would consider their cultural context and available resources. Enhancing the effectiveness of patient self-initiated strategies can be achieved through well-structured interventions that target physical and mental fatigue among patients receiving hemodialysis.

## Electronic supplementary material

Below is the link to the electronic supplementary material.

Supplementary Material 1

## Data Availability

The datasets used and analyzed during the current study are available from the principle author on reasonable request.

## Abbreviations

ESKD: End-stage kidney disease
RRT: Renal replacement therapy
PDF: Post dialysis fatigue
HIV: Human immunodeficiency virus
HCPS: Healthcare providers
